# Identification of BLCAP as a novel STAT3 interaction partner in bladder cancer

**DOI:** 10.1371/journal.pone.0188827

**Published:** 2017-11-30

**Authors:** Irina Gromova, Sofia Svensson, Pavel Gromov, José M. A. Moreira

**Affiliations:** 1 Cancer Proteomics, Genome Instability Unit, Danish Cancer Society Research Center, Copenhagen, Denmark; 2 Department of Drug Design and Pharmacology, Faculty of Health and Medical Sciences, University of Copenhagen, Copenhagen, Denmark; Biomedical Research Foundation, Academy of Athens, GREECE

## Abstract

Bladder cancer associated protein (Blcap) expression is commonly down-regulated in invasive bladder cancer, and may have prognostic value given that its expression is negatively correlated with patient survival. We have previously investigated the expression patterns and cellular localization of Blcap in bladder cancer, where we found that about 20% of the lesions examined displayed strong nuclear expression of Blcap, and that this phenotype was associated with overall poor disease outcome. Here we report on the analysis of possible functional associations between nuclear expression of Blcap and canonical signaling pathways. We performed serial immunohistochemistry (IHC) analysis of bladder tissue samples, with serial sections stained with phospho-specific antibodies recognizing key signaling intermediates, such as P-Stat3, P-Akt, and P-Erk1/2, among others, in an immunophenotyping approach we have established and reported previously. Using this approach, we found that nuclear localization of Blcap was associated with expression of P-Stat3. A parallel analysis, cytokine profiling of bladder tumor interstitial fluids of samples expressing (or not) Blcap, showed interleukin (IL)-6, IL-8, and monocyte chemotactic protein 1 (MCP-1) to be correlated with nuclear expression of Blcap, independently supporting a role for Stat3 signaling in localization of Blcap. Multiple indirect immunofluorescence analysis of tissue biopsies confirmed that Blcap co-localized with Stat3. Furthermore, we could also demonstrate, using an in situ proximity ligation assay that Blcap and Stat3 are in close physical proximity of each other in bladder tissue, and that Blcap physically interacts with Stat3 as determined by co-immunoprecipitation of these proteins. Our data indicates that Blcap is a novel Stat3 interaction partner and suggests a role for Blcap in the Stat3-mediated progression of precancerous lesions to invasive tumors of the bladder.

## Introduction

Bladder Cancer Associated Protein (Blcap), is a small (10 kDa), highly conserved protein whose expression is lost in various cancers, such as cervical, bladder and renal cancer, as well as in human tongue carcinoma and osteosarcoma [1–7]. Data from our laboratory has also shown that in bladder cancer, tumor progression is generally associated with loss of expression of Blcap [1, 2]. Over-expression of *BLCAP* in human TC-135 Ewing’s sarcoma cells, Tca8113 tongue carcinoma cells, and HeLa cervical cancer cells can inhibit cell growth and induce apoptosis [4, 8, 9], suggesting that Blcap may regulate cancer cell proliferation and survival, and play a role in cellular carcinogenesis.

We have previously investigated the expression of Blcap in bladder cancer in a set of 120 bladder tissue specimens [1]. We found that Blcap was expressed in urothelial cells, with weak to moderate cytoplasmic staining and strong irregular nuclear staining. We have also shown that in some cases, however, Blcap is over-expressed and tumors that show strong nuclear expression are linked with poor disease outcome, suggesting that expression of Blcap confers an adverse patient outcome [1]. The association we identified suggested a link between nuclear expression of Blcap and disease outcome, but the mechanism(s) underlying this phenomenon are unknown. Matching of tumor samples with corresponding benign specimens collected from the same patient, showed that although loss of Blcap expression in tumor cells was a common event, in roughly 25% of the cases, Blcap was strongly up-regulated with marked nuclear expression [1]. In addition, patients bearing tumors with increased nuclear expression of Blcap had a worse outcome. Given that Blcap is reportedly a tumor suppressor, able to inhibit cell proliferation and induce apoptosis [4, 9], it was somewhat counterintuitive that some tumors expressed this protein at very high levels, and that overexpression conferred a worse prognosis. Another challenging observation we made, concerned the strong nuclear Blcap expression observed, because primary sequence analysis of Blcap using two different protein topology prediction methods indicated Blcap as being an integral transmembrane protein (total probability of N-in 0.087213 for TMMOD and 0.01091 for TMHMM), with two trans-membrane domains, TM^20-38^ and TM^45-69^, respectively [1]. Yet, we found it to be present in the cytoplasm and nucleus, which was suggestive of an active transport/localization event. To investigate the biological underpinnings of these observations, we set out to identify factors involved in Blcap overexpression and/or nuclear localization.

Here we identify Signal transducer and activator of transcription 3 (Stat3)e as a Blcap interacting partner in bladder cancer and show that Blcap nuclear expression is associated with Stat3 expression. Stat3, is one out of seven members of the signal transducer and activator of transcription (STAT) family of transcription factors, a family of proteins which has been found to be constitutively activated in numerous cancer types. Stat3 transduces cytokine and growth factor signaling in cells, transcriptionally regulating a diverse array of cellular processes germane to cancer, such as cell proliferation, apoptosis, angiogenesis, immune response and metastasis. Stat3 is a transcription factor that promotes the progression of urothelial cells from carcinoma in situ to invasive bladder cancer [10, 11]. Activation of Stat3 by upstream kinases is dependent on phosphorylation of a tyrosine residue at position 705, with ensuing dimerization and nuclear translocation [12]. Stat3 phosphorylation and consequent activation can occur in response to a vast range of extracellular stimuli, including growth factors and cytokines such as IL-6 [12]. Activation of Stat3 can promote cell survival and proliferation, and it has been linked to malignant transformation.

Our findings propose Blcap as a novel interactor of Stat3 in bladder cancer and, possibly, as a factor involved in bladder cancer progression through the Stat3 signaling pathway.

## Methods

### Bladder tissue specimens

Formalin fixed paraffin-embedded (FFPE) tissue random biopsies, consisting of histologically normal tissue specimens and urothelial carcinomas of various grades and stages, collected over a period of 6 years at Skejby Hospital, Aarhus, Denmark were analyzed. Tumors were classified by an experienced pathologist according to Bergkvist and colleagues [13]. All samples were from the MOB bladder tissue research biobank, which is part of the Danish Cancer Biobank (www.cancerbiobank.dk). The study was approved by the Central Denmark Region Committees on Biomedical Research Ethics (#1994/2920). All research was conducted according to the principles expressed in the Declaration of Helsinki, with written informed consent obtained for each patient. No minors were included in the study.

### Cell culturing

The human urinary bladder carcinoma T24 cell line was purchased from the American Type Culture Collection (ATCC, Rockville, MD, USA) and cells were cultured in McCoy’s 5a modified medium (Invitrogen, Carlsbad, CA, USA) supplemented with 10% heat-inactivated FCS (Sigma-Aldrich, USA). Cells were incubated at 37°C, under 5% CO_2_ controlled atmosphere in a humidified incubator.

### 2D PAGE and 2D western immunoblotting

2D PAGE gels and 2D Western immunoblotting were performed as previously described [1].

### Interstitial fluid and cytokine arrays

Bladder tumor interstitial fluids were recovered from fresh bladder tissue biopsies as previously described [14, 15]. Briefly, for each sample, approximately 0.1–0.3 g of clean tissue was cut into small pieces (~1 mm^3^ each), washed twice in cold PBS to remove blood and cell debris, and then incubated in PBS for 1 h at 37°C in a humidified CO_2_ incubator. The samples then were centrifuged at 1,000 rpm and 5,000 rpm for 2 min and 20 min, respectively, at 4°C. After the supernatants were carefully aspirated, total protein concentration for each sample was determined with the Bradford assay. Detection of cytokines present in bladder TIF was done using antibody array-based technology (RayBio^™^ Cytokine Antibody Arrays 5.1, RayBiotech, Inc., Atlanta, USA). We acquired membrane-bound antibody arrays and profiled cytokine content in bladder TIF from four different tumor biopsies with varying nuclear expression of Blcap. Each array was incubated with 0.5 ml of diluted TIF (1mg/ml final concentration) at 4°C overnight, and bound cytokines were detected according to manufacturer’s instructions. Samples were normalized based on total protein content, which in turn was derived from 2D PAGE gel images of the samples. Protein concentrations in all cases were between 1-4mg/ml.

### Immunohistochemistry

Immunohistochemistry assays were performed using tissue sections of paraffin-embedded samples essentially as described [1]. Briefly, 5-μm tissue sections were mounted on Super Frost Plus slides (Menzel-Gläser, Braunschweig, Germany), deparaffinized and rehydrated through graded alcohol rinses. Heat induced antigen retrieval was performed by immersing the slides in and heating them for 10 min in a 750 W microwave oven. Non-specific staining of slides was blocked with 1% FCS for 15 min, and endogenous peroxidase activity quenched with 0.3% H_2_O_2_ for 30 min. Antigen detection was done by incubation first with a relevant primary antibody, followed by a species matched secondary antibody conjugated to a peroxidase complex (Envision+ poly-HRP system, DAKOCytomation, Denmark). Color development was done using DAB+ Chromogen, (DAKOCytomation, Denmark). Slides were counterstained with hematoxylin. Antibodies used are listed in S1 Table.

### Indirect immunofluorescence analysis

For colocalization studies we performed immunofluorescence analysis of FFPE sections essentially as described [16]. Briefly, 4-μm sections cut from FFPE blocks of bladder tissue samples were mounted on Super Frost Plus slides (Menzel-Gläser, Braunschweig, Germany), deparaffinised, and rehydrated through graded alcohol rinses. Heat-induced antigen retrieval was carried out as described above. Following antigen retrieval, sections were treated with Image-iT FX^™^ signal enhancer (Molecular Probes, OR, USA) to block non-specific staining and subsequently incubated with the relevant primary antibodies at the appropriate dilution. Secondary antibodies conjugated to Alexa Fluor^®^ 488, and Alexa Fluor^®^ 568 (Molecular Probes) were used for detection of immune complexes. Nuclear material was counterstained with DAPI. Sections were imaged using a Zeiss LSM510META confocal laser scanning microscope (Carl Zeiss MicroImaging Gmbh, Germany).

### In situ proximity ligation assay

Proximity of Blcap and Stat3 proteins was investigated using Duolink In Situ (OLINK Bioscience, Sweden) according to manufacturer’s instructions. Briefly, FFPE tissue sections were deparaffinized and rehydrated through alcohol rinses. Heat-induced antigen retrieval was done in Tris/EDTA pH 9.0 buffer (10mM Tris, 1mM EDTA). The sections were sequentially incubated with a rabbit anti-Blcap antibody (1:100) (Eurogentec, Seraing, Belgium) and a monoclonal mouse anti-Stat3 antibody (1:150) (Cell Signaling, Cell Signaling Technology, MA, USA) for 60 min each, respectively. Species specific secondary antibodies linked to specific oligonucleotides (PLA probes) were added, and the sections were incubated for an additional 60 min at 37°C. Ligation of PLA probes was done by adding a solution containing ligase to tissue sections at 37°C for 15 min. Finally, signal amplification took place by rolling circle amplification of ligated PLA probes at 37°C for 90 min. Sections were stained with DAPI to visualize cell nuclei. Addition of isotype control immunoglobulins instead of primary antibodies was used as negative control. Cells were imaged using a Zeiss LSM510META confocal laser scanning microscope (Carl Zeiss MicroImaging Gmbh, Germany). For each sample, five different areas were examined.

### Co-immunoprecipitation analysis

For Stat3 and Blcap co-immunoprecipitation, T24 bladder carcinoma cells were used. Cells were cultured in McCoy’s 5a modified medium (Invitrogen, Carlsbad, CA, USA) supplemented with 10% heat-inactivated FCS until they reached 70% confluency, they were then serum-starved for 1h and subsequently stimulated or not stimulated with IL-6 (20ng/ml) for 6 h to induce activation and nuclear translocation of Stat3. Cells were washed twice with ice-cold PBS and lysed with lysis buffer (25 mM Tris, 150 mM NaCl, 1 mM EDTA, 1% NP-40, 5% glycerol, pH 7.4) containing phosphatase and protease inhibitor mixtures (Roche Applied Science). For cell lysis and fractionation of cytoplasmic and nuclear protein extracts, the NE-PER Nuclear and Cytoplasmic Extraction Kit was used according to manufactorer’s instructions (Thermo Fischer Scientific, USA). For immunoprecipitation of proteins, lysates were precleared for 2h with Protein G-Dynabeads (Invitrogen) to minimize unspecific binding and then incubated overnight with a Stat3 antibody (Cell Signaling Technology, MA, USA) or isotype control pre-bound to Protein G-Dynabeads. The resin was then washed four times with ice-cold lysis buffer. Beads were resuspended in 30 μl of Laemmli sample buffer, boiled for 3 min and centrifuged at 14 000 g for 5 min. The supernatants were analysed by immunoblotting as described [1].

## Results and discussion

### Blcap expression and localization is correlated with that of Stat3

The interspersed nuclear staining of Blcap we observed in our previous work, with some cells showing presence of this protein in the cytoplasm, but not in the nucleus, suggested to us that localization of this protein to the nucleus might be a regulated function. This notion was reinforced by the fact that protein topology prediction methods identified two transmembrane domains in Blcap, but we also observed nuclear and cytoplasmic localization [1]. Consequently, we hypothesized that Blcap may localize to the nucleus in response to effectors present in the microenvironment or intracellular signaling events. To address which environmental cues and signaling events were associated with nuclear localization of Blcap, we employed a parallel two-sided experimental approach, profiling cytokines present in the environment of bladder samples and simultaneously profiling signaling pathways in bladder tissue samples.

#### 1.Cytokine profiling

**Fig 1:**
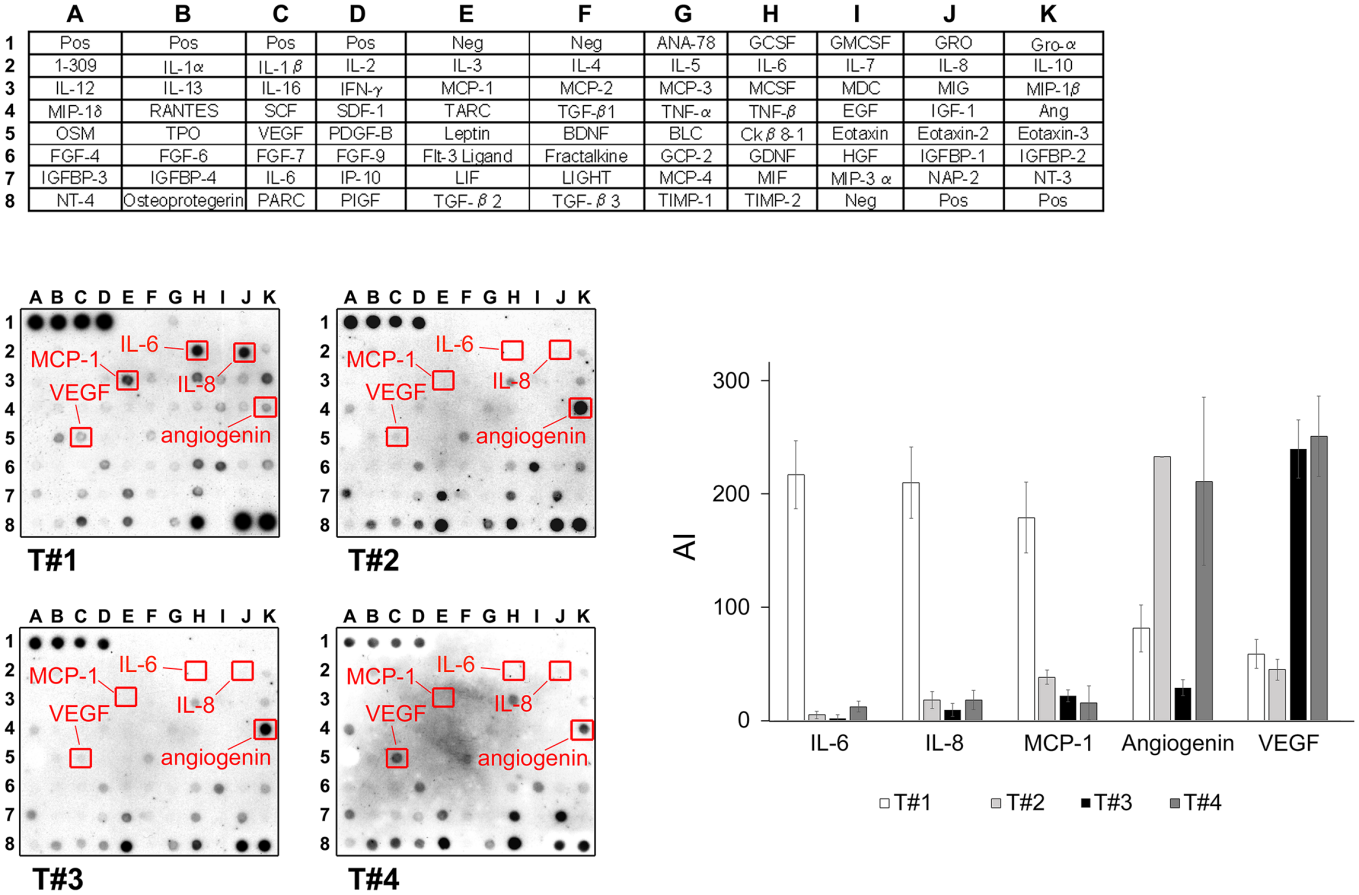
Cytokine profiling of bladder tumor interstitial fluid. Cytokine-specific antibody arrays (RayBio^®^ human cytokine array 5.1, RayBioTech Inc, USA) were incubated with 0.5 ml of TIF from four different samples either strongly expressing Blcap (T#3), or devoid of Blcap (T#4, T#5, and T#6), respectively, according to the manufacturer’s instructions. Expression of three cytokines, interleukin (IL)-6, IL-8, and monocyte chemotactic protein 1 (MCP-1), correlated with the Blcap expression status of the samples. Two other factors, VEGF and angiogenin, are also highlighted as examples of seemingly uncorrelated factors predominantly expressed in one of the samples. A map of the cytokines detected by the antibody array is provided on the upper panel.

Cytokines and growth factors present throughout the tumor interstitial fluid (TIF) environmental cues to tumors, mediate multidirectional signaling events between cancer cells and the stromal microenvironment, thus modulating the progression of malignant cells. Given the possibility that nuclear presence of Blcap in urothelial carcinomas (UCs) was a cellular response to environmental cues, we performed array-based expression profiling of cytokines and growth factors present in the microenvironment of UCs, and correlated these with intracellular Blcap expression patterns in corresponding samples. To do this we took advantage of a technique developed in our laboratory that allows the collection of the interstitial fluid, which contains all molecules secreted and externalized from the cells in a given tumor mass (tumor and its microenvironment) [14]. Accordingly, we collected and profiled the cytokine content of interstitial fluid from four malignant bladder tissue samples that displayed different levels of Blcap expression, and for which we had sufficient biological material to do this analysis. One sample had strong Blcap nuclear expression (T#3), whereas the other three samples had low or no detectable overall immunoreactivity for Blcap (T#4, T#5, and T#6). We collected TIF from these samples as previously described and We used antibody cytokine arrays (RayBio^™^ Cytokine Antibody Arrays 5.1, RayBiotech, Inc., Atlanta, USA) that allow one to monitor the abundance of 78 human cytokines simultaneously. Quantitative comparison of these cytokines among the different samples showed a correlation between the secreted level of three cytokines, interleukin (IL)-6, IL-8, and monocyte chemotactic protein 1 (MCP-1), and nuclear Blcap expression status (Fig 1, rightmost panel). The specimen that had nuclear expression of Blcap showed high levels of IL-6, IL-8, and MCP-1 whereas the three specimens with low/absent expression of Blcap had low or undetectable levels of these cytokines (Fig 1), suggesting that one or more of these cytokines may be associated with Blcap nuclear expression status.

#### 2. Signaling profiling

**Fig 2:**
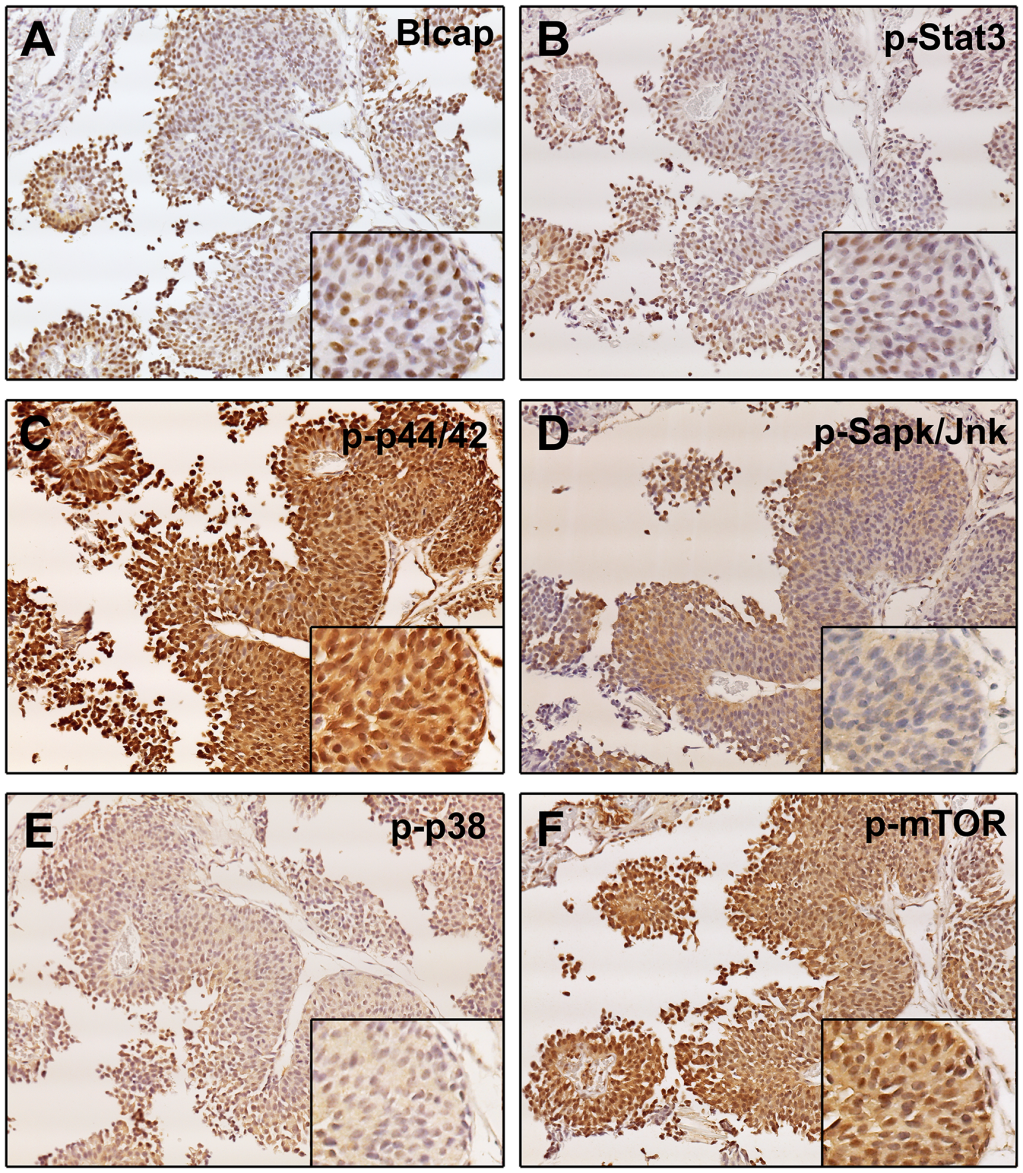
Immunophenotyping of Blcap positive tumor cells. Tandem sections of a bladder tumor specimen (T#9) were stained with antibodies against Blcap, p-Stat3, p-mTor, p-p44/42, p-Sapk/Jnk, and p-p38 and allowing us to determine which signaling event was associated with Blcap expression. Magnification, 20x. Inset are higher magnifications (40x) of the same region in the consecutive sections.

The phosphorylation state of key regulators in a given kinase-driven signal transduction pathway is generally used as a measure of the activity status of that particular pathway, and consequently of its contribution to a given cell’s response to different environmental stimuli. To investigate which signaling events, if any, were associated with nuclear expression of Blcap, we used an immunophenotyping approach that enables one to characterize the molecular phenotypes of cells of interest—a method that we have previously used to find correlations between molecular traits in tissue samples [16]. Serial sections were analyzed by immunohistochemistry using antibodies specific for the active protein forms of several major regulatory hubs in cell signaling. We used antibodies recognizing key factors involved in signal transduction, AKT signaling, cell cycle / DNA damage, apoptosis, translational, Jak/Stat pathway, proliferation, and tyrosine kinase (TK) receptor signaling (S1 Table). Correlation of the activation state and expression of these key cellular regulators with Blcap expression was assessed by a combined approach, using IHC and multiple indirect-label immunofluorescence analysis of serial sections of bladder samples. We analysed serial sections of a selected subset of 16 samples, which had shown strong nuclear expression of Blcap and for which we had sufficient biological material to perform this analysis, from our reference set of 120 bladder specimens (illustrated in Fig 2, panels A through F). We found that of the total of 21 proteins examined in this manner, only one single marker, phospho-Stat3 (Tyr^705^) (p-Stat3), showed the same speckled immunoreactivity we observed for Blcap (illustrated in Fig 2, compare panels A and B, respectively). For all other cases either we saw general lack of immunoreactivity for the signaling factor (illustrated in Fig 2, compare panels A and C, respectively) or general overall expression of the signaling factor (illustrated in Fig 2, compare panels A and C, respectively).

**Fig 3:**
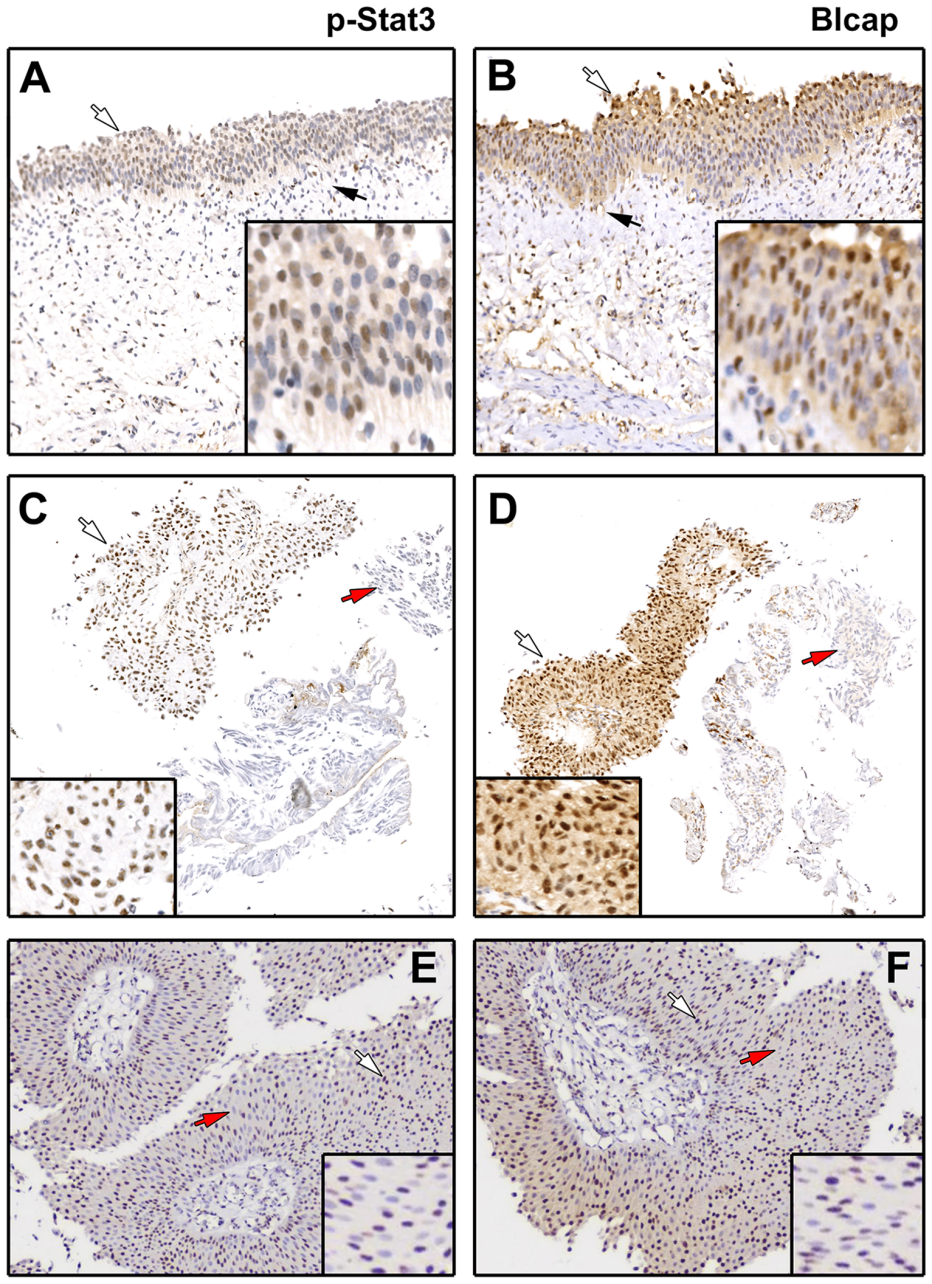
Staining patterns of Blcap and p-Stat3. (A and B) IHC staining of Blcap and p-Stat3, respectively, in consecutive sections of a non-malignant bladder section demonstrated the expression of the two antigens in urothelial cells with weak cytoplasmic and moderate, scattered nuclear expression. Black arrow points to a region showing expression of Blcap. (C and D) IHC of an UC (T#10) composed of two distinct lesions, one with strong expression of Blcap (black arrow) and another with no expression of this protein (red arrow), displayed the exact same staining pattern for p-Stat3, with the same areas being positive (black arrow) or negative (red arrow) for p-Stat3, respectively. (E and F) an UC sample (T#1) with a very distinctive Blcap interspersed nuclear staining showed an almost identical staining pattern for p-Stat3, with positive (black arrow) and negative cells (red arrow) intermingling. Inset are higher magnifications (40x) of selected representative areas of each section.

Patterns of Blcap expression were nearly identical to those of p-Stat3 in all 16 samples examined. We then extended our analysis to the other bladder specimens we had, including non-malignant biopsies (*n* = 24) and UCs (*n* = 80) (Fig 3). Again, we found that the patterns of expression of Blcap were similar to those of p-Stat3, although not identical: in non-malignant specimens we observed that both p-Stat3 and Blcap localized predominantly to the urothelium, with weak to moderate cytoplasmic staining and interspersed moderate to strong nuclear staining of urothelial cells (Fig 3, compare panels A and B, white arrows). In tumor samples this similarity of staining patterns between p-Stat3 and Blcap was also evident (Table 1 and Fig 3, compare panels 3C with 3D, and 3E with 3F, respectively).

**Table 1:** Correlation between Blcap and p-Stat3 expression in 80 UC samples.

Sample	Grade	Stage	Blcap*	p-Stat3**
1	G1	Ta	-	-
2	G1	Ta	-	-
3	G1	Ta	-	+
4	G1	Ta	-	-
5	G1	Ta	-	-
6	G1	Ta	-	-
7	G1	Ta	-	+
8	G1	Ta	-	++
9	G1	Ta	+	-
10	G1	Ta	+	-
11	G1	Ta	+	+
12	G1	Ta	++	+
13	G1	Ta	++	++
14	G1	Ta	++	++
15	G1	Ta	++	++
16	G1	Ta	++	++
17	G1	Ta	++	++
18	G1	Ta	++	++
19	G1	Ta	-	-
20	G2	T1	++	+
21	G2	T1	++	++
22	G2	Ta	-	-
23	G2	Ta	-	-
24	G2	Ta	-	-
25	G2	Ta	+	++
26	G2	Ta	++	+
27	G2	Ta	++	+
28	G2	Ta	++	+
29	G2	Ta	++	++
30	G2	Ta	++	++
31	G2	Ta	++	++
32	G2	Ta	++	++
33	G2	Ta	++	++
34	G2	Ta	++	++
35	G2	Ta	++	++
36	G2	Ta	++	++
37	G2	Ta	++	++
38	G2	Ta	++	++
39	G2	Ta	++	++
40	G3	T1	++	++
41	G3	T1	-	-
42	G3	T1	-	-
43	G3	T1	-	++
44	G3	T1	-	++
45	G3	T1	+	+
46	G3	T1	+	+
47	G3	T1	+	+
48	G3	T1	++	+
49	G3	T1	++	++
50	G3	T1	++	++
51	G3	T1	++	++
52	G3	T2-4	-	-
53	G3	T2-4	-	-
54	G3	T2-4	-	-
55	G3	T2-4	-	-
56	G3	T2-4	-	+
57	G3	T2-4	+	-
58	G3	T2-4	++	++
59	G3	T2-4	-	-
60	G3	T2-4	-	-
61	G3	T2-4	-	-
62	G3	T2-4	-	-
63	G3	T2-4	-	+
64	G3	T2-4	-	+
65	G3	T2-4	++	+
66	G3	T2-4	++	+
67	G3	T2-4	++	+
68	G3	T2-4	++	+
69	G3	T2-4	++	++
70	G3	T2-4	++	++
71	G3	T2-4	++	++
72	G3	Ta	-	++
73	G3	Ta	+	+
74	G3	T1	+	+
75	G4	T2-4	-	-
76	G4	T2-4	+	-
77	G4	T2-4	-	-
78	G4	T2-4	-	-
79	G4	T2-4	-	+
80	G4	T2-4	-	+

*Blcap staining was scored as strong nuclear (++), weak nuclear (+), or no nuclear staining (-)

**p-Stat3 staining was scored as strong nuclear (++), weak nuclear (+), or no staining (-)

**Fig 4:**
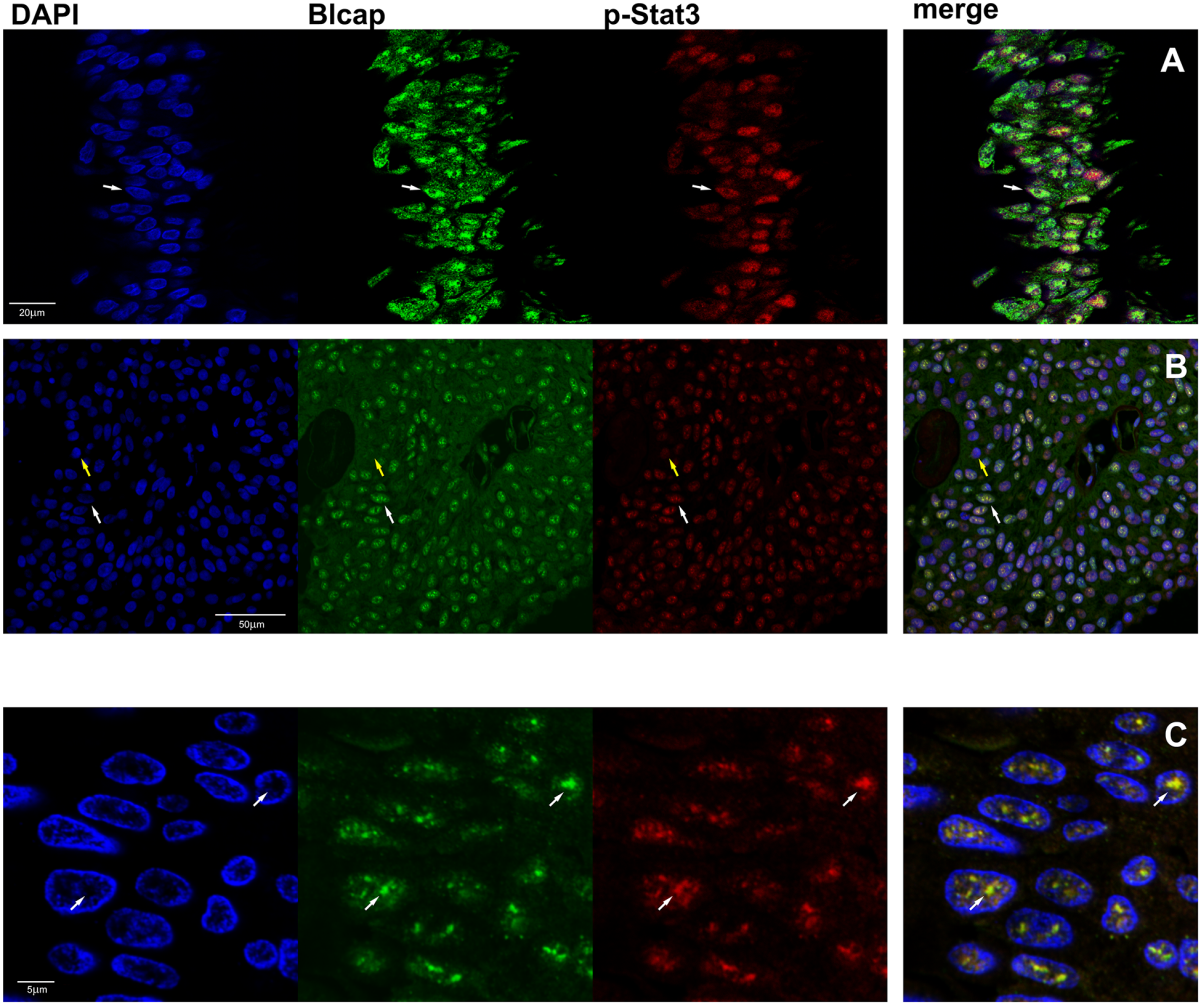
Colocalization of Blcap and p-Stat3. Indirect double label immunofluorescence analysis of tissue sections incubated with Blcap (Alexa Fluor 488; green) and p-Stat3 (Alexa Fluor 594; red channel) antibodies and counterstained with the nuclear stain DAPI (blue). In the merged image, co-localization of both antigens shows as yellow. (A) benign specimen showing co-localization of Blcap and p-Stat3 in urothelial cells with nuclear presence of both proteins (white arrow). (B) in a malignant specimen (T#1) with strong scattered nuclear expression of Blcap and p-Stat3, cells either expressed both proteins (white arrow) or neither one (yellow arrow). (C) Higher magnification of the malignant specimen T#1, showed that Blcap localized to well defined bodies within the nucleus and the p-Stat3 was present in the same bodies (white arrows).

To confirm co-localization of these two proteins, and since the interspersed character of the nuclear expression of p-Stat3 and Blcap (Fig 3E and 3F, respectively), made it difficult to ascertain whether the correlation was positive or negative, we investigated the localization of these two proteins by multicolor immunofluorescence analysis (Fig 4).

We found that p-Stat3 and Blcap co-localized in samples with strong nuclear staining of Blcap, both in non-malignant (Fig 4A, white arrows) and tumor samples (Fig 4B) displaying strong interspersed nuclear localization of the two proteins. Co-localization of the two proteins is rather striking, with tumor cells devoid of nuclear Blcap expression also not showing p-Stat3 (Fig 4B, yellow arrow). As reported previously, we found that Blcap was diffusely distributed throughout the nucleoplasm with a strong, irregular, punctate fluorescent pattern that did not include the nucleoli. The punctate pattern observed for Blcap was closely matched by p-Stat3 (Fig 4C, white arrows), establishing that Stat3 and Blcap co-localized.

**Fig 5:**
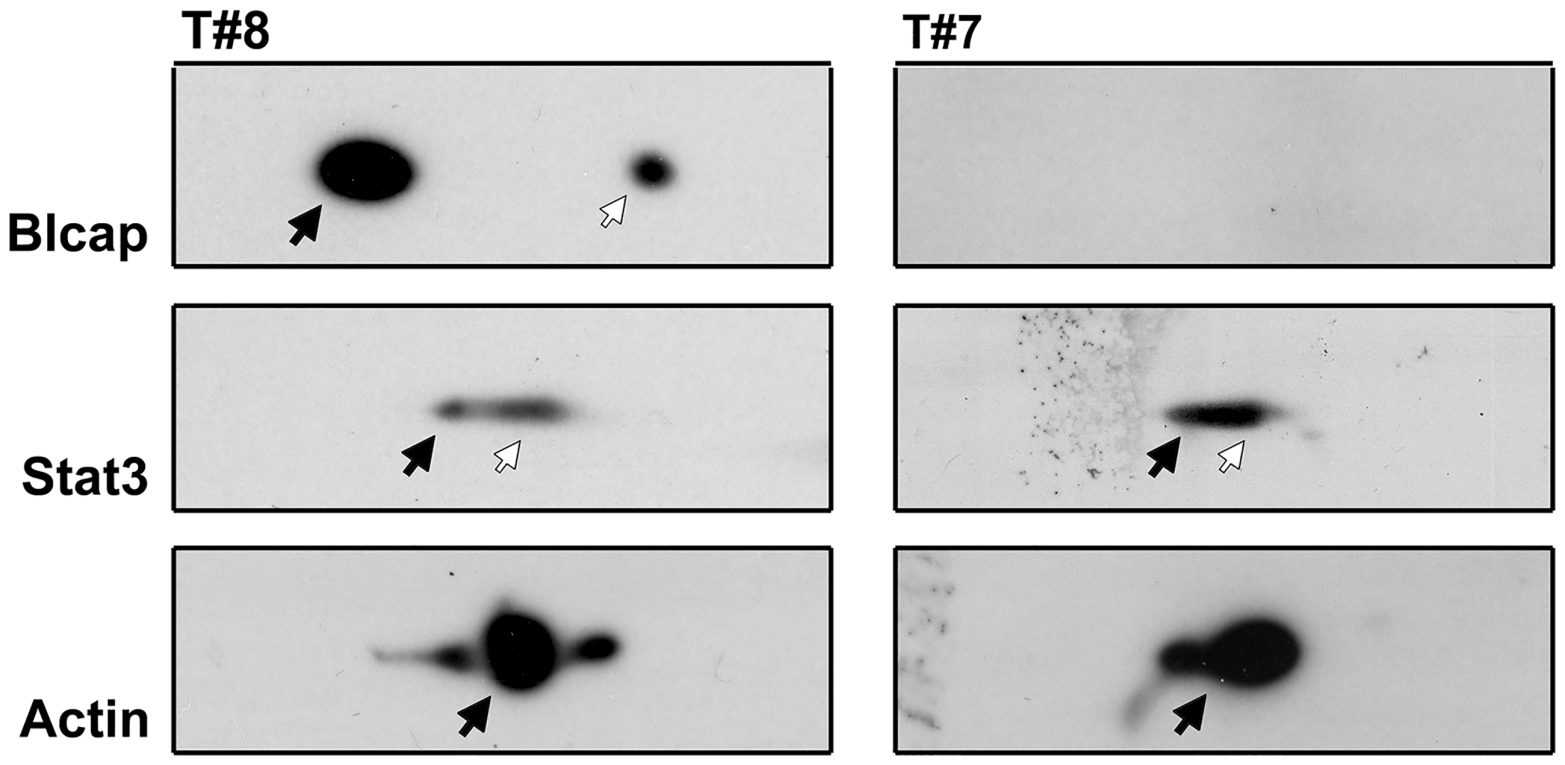
2D immunoblotting analysis. Lysates of two tissue specimens determined by IHC to express Blcap (A) or not (B) were resolved by 2D PAGE (IEF) and blotted onto a nitrocellulose membrane. The immunoblot protein patterns show that expression of Blcap followed the IHC analysis and that Stat3 was expressed at similar levels in both cases. Black arrows indicate the primary form- and white arrows a posttranslational modified form of the protein, presumably due to phosphorylation as previously described [1]. Actin was used as loading control.

Although we found that Blcap co-localized with p-Stat3, expression of the two proteins was not strictly correlated, and in several cases we found that samples devoid of Blcap exhibited strong expression of p-Stat3 (Table 1 and Fig 5).

The converse also occurred, with tumors showing Blcap expression and no detectable p-Stat3. Of note, we did not discover a single case with strong nuclear expression of Blcap and no nuclear expression of p-Stat3. Because it was conceivable that lack of immunoreactivity for Blcap was due to epitope accessibility issues, we performed 2D western immunoblotting for samples displaying strong immunoreactvity for p-Stat3 and Blcap, or with strong immunoreactvity for p-Stat3 but no Blcap staining (Fig 5). The western blot analysis reiterated the IHC results confirming that lack of reactivity for Blcap just reflected lack of protein expression. We concluded that expression of Blcap was associated with that of Stat3, but that mere expression and activation of Stat3 was not sufficient to induce Blcap expression.

### Blcap interacts with Stat3

**Fig 6:**
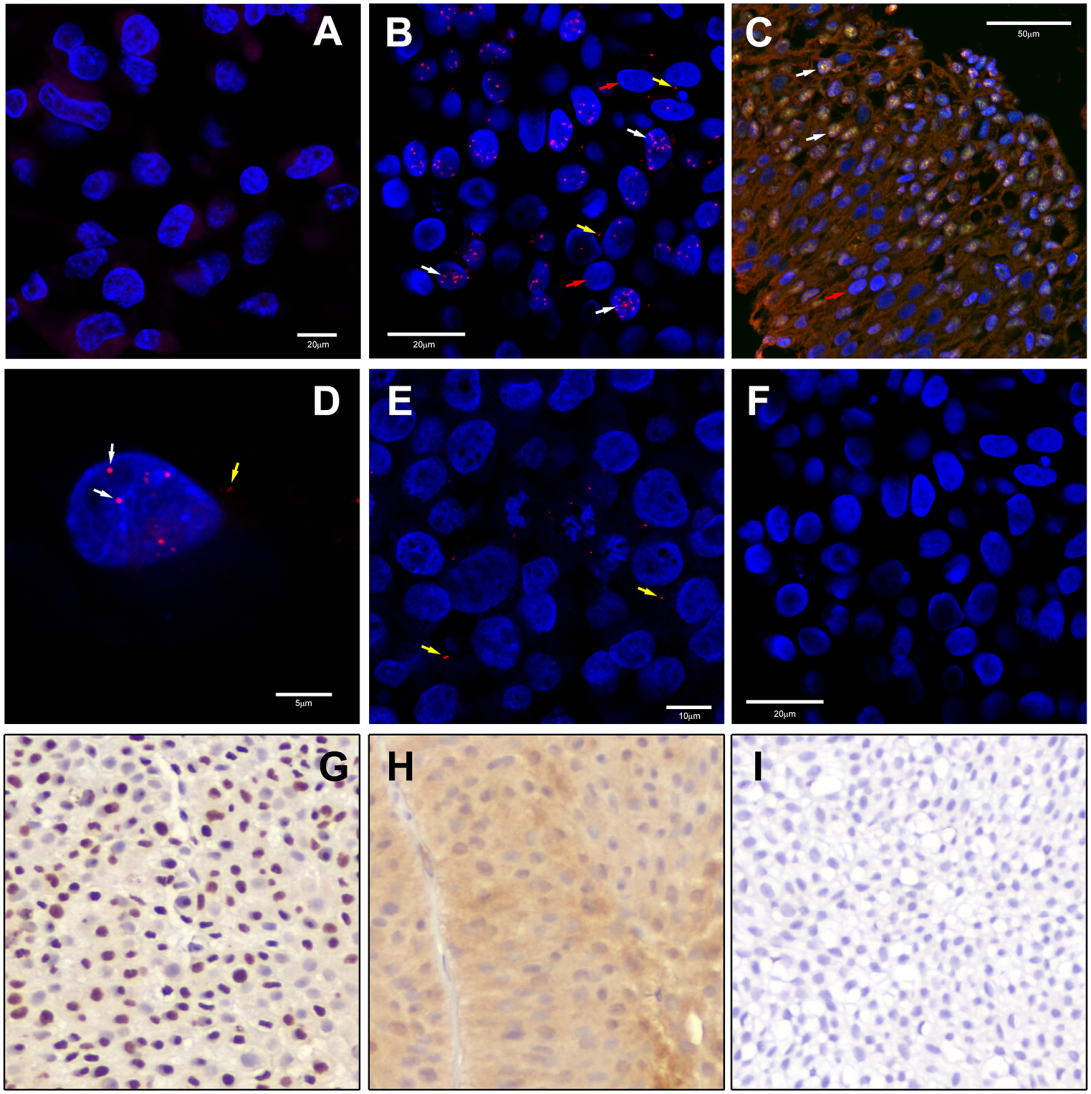
Proximity ligation assay. Representative images for Blcap and Stat3 PLA assay in bladder cancer samples showing nuclear staining (DAPI) in blue and PLA signals in red. (A) For control experiments, one of the primary antibodies was exchanged for an isotype-matched control antibody. (B) PLA signals in a sample (T#2) with strong scattered nuclear expression of Blcap and Stat3, show that the two proteins are in close proximity of each other (white arrows) in some nuclei, and in more spuriously in the cytoplasm (yellow arrows). Other nuclei had no detectable PLA signals (red arrows). This pattern was consistent with immunofluorescence results for a tandem section (C) that showed colocalization of Blcap and Stat3 in some nuclei (white arrows) and no expression of either protein in other nuclei (red arrow). (D) Higher magnification clearly shows the nuclear PLA signals (white arrow) and occasional cytoplasmic signal (yellow arrow). (E) PLA analysis of a sample that showed moderate cytoplasmic expression of Blcap (panel H) displaying only occasional cytoplasmic PLA signals. (F) PLA analysis of a sample that had no detectable expression of Blcap also lacked PLA signals. (G-I) IHC images of samples used for PLA analysis of panels B, E, and F, respectively.

Having shown that Blcap co-localized with p-Stat3, we were interested in determining whether this was a fortuitous event, with both proteins simply residing in the same nuclear compartment, or if Blcap physically interacted with Stat3. We used *in situ* Proximity Ligation Assay (PLA), a method allowing one to visualize protein-protein interactions in tissue sections [17, 18], to determine whether Blcap was in physical proximity of Stat3 in bladder cancer samples (Fig 6).

In samples with strong nuclear expression of Blcap and Stat3 (Fig 6C and 6G), we detected a number of fluorescent signals in the presence of both antibodies, indicative of an interaction between Blcap and Stat3 in the nucleus (Fig 6B), whereas no signals were detected in the absence of one of the antibodies (Fig 6A, negative control) or in samples with cytoplasmic, but not nuclear, expression of Blcap (Fig 6E and 6H) or devoid of Blcap altogether (Fig 6F and 6I). We concluded, based on these results that Blcap interacts with Stat3 in the nuclei of bladder cancer cells.

**Fig 7:**
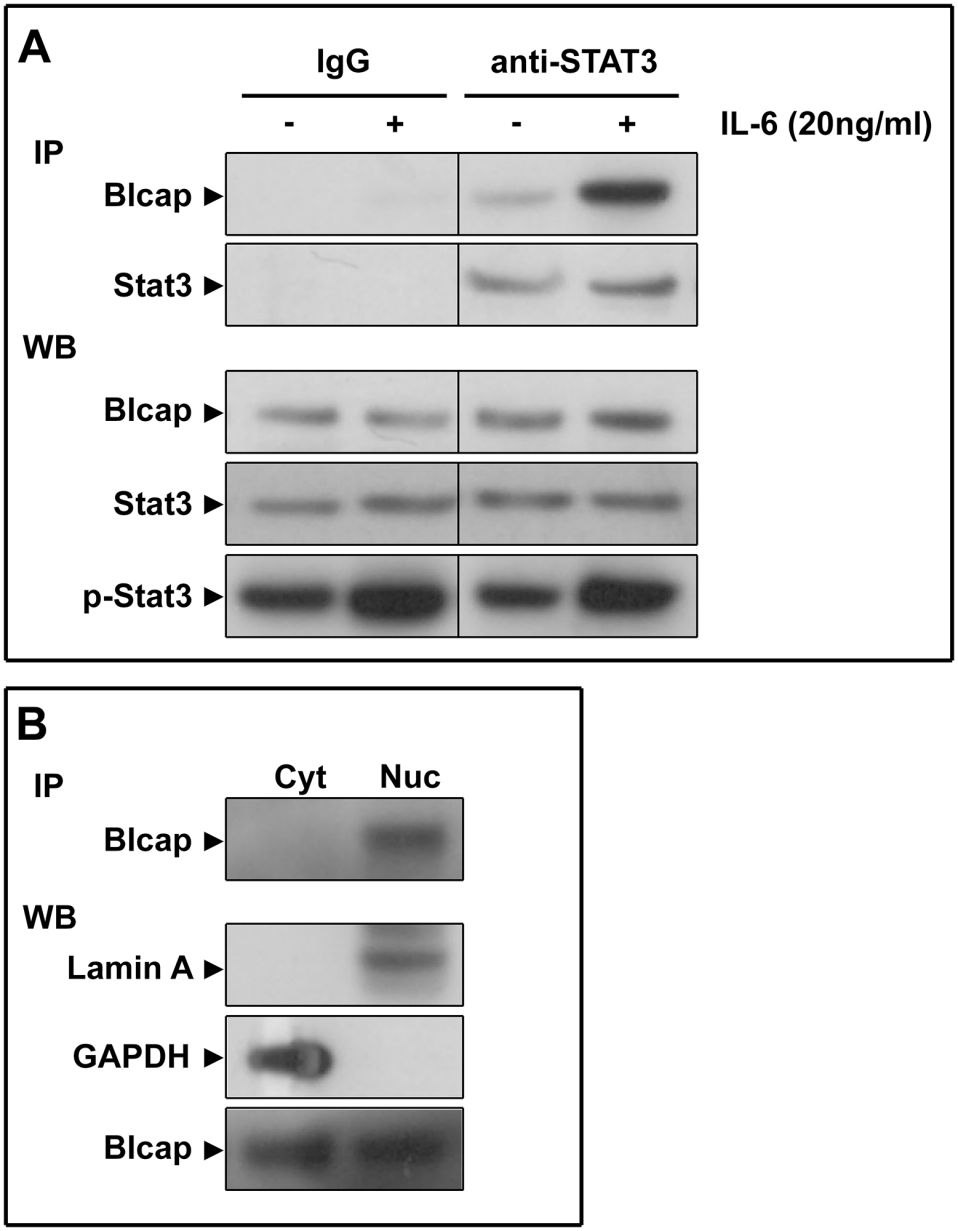
Coimmunoprecipitation analysis. (A) Lysates of T24 cells stimulated (+) or not (-) with IL-6 (20ng/ml) were immunoprecipitated either with an antibody against Stat3 (anti-Stat3), or an IgG isotype control antibody (IgG). Pulled-down proteins were probed for presence of Stat3 and Blcap (IP; upper panels). Western blot analsysis of lysates showed that neither Blcap nor Stat3 expression did not change markedly with exposure to IL-6 (lower panels), but that Stat3 got phosphorylated upon cell stimulation with IL-6. (B) Immunoprecipitation with an antibody against anti-Stat3 of cytoplasmic (Cyt) or nuclear (Nuc) fractions from IL-6 stimulated T24 cells only showed presend of Blcap in the nuclear fraction. Western blot analysis of extracts with antibodies against Lamin A and GAPDH were used as nuclear or cytoplasmic markers, respectively.

To verify the results we obtained with the *in situ* PLA analysis, we carried out co-immunoprecipitation experiments with antibodies against Stat3, in cell lysates prepared from T24 bladder cancer cells (Fig 7).

When the lysates were subjected to immunoprecipitation, we detected Blcap in the immunoprecipitates pulled down with an anti-Stat3 antibody, which indicates that Blcap is capable of interacting with Stat3 in T24 cells. This interaction was increased upon stimulation with IL-6 (Fig 7; pull-down with Stat3, +IL-6) when compared to non-stimulated cells (Fig 7A; pull-down with Stat3, -IL-6), suggesting that activation of Stat3 potentiates the interaction with Blcap. Stimulation of T24 bladder cancer cells with IL-6, leads to Stat3 activation, as determined by phosphorylation of Stat3 (Fig 7A, western blot, p-Stat3), but we found no evidence of increased Blcap expression (Fig 7A, western blot, Blcap). To determine whether the interaction was spatially restricted, we performed immunoprecipitation of nuclear and cytoplasmic fractions with an anti-Stat3 antibody, and found Blcap interacted with Stat3 in the nuclear fraction, but were unable to find an interaction in the cytoplasmic fraction (Fig 7B).

In short, from our interaction data, PLA and immunoprecipitation analysis, it would follow that Blcap interacts with Stat3. Stat3 is a Src homology (SH)2-domain containing protein that is recruited to the cytoplasmic domain of cytokine and growth factor receptors via the SH2 domain [19]. The SH2 domain of Stat3 is recruited to cognate factors through a docking site with a YXXQ consensus motif. We did a sequence analysis of Blcap and identified a YXXQ motif, Y^2^CLQ^5^ proximal to one of the transmembrane domains, TM^20-38^, a situation reminiscent of what is seen in a variety of signal-transducing receptors, including gp130, LIF-R, G-CSFR, leptin-R, and IL10-R [19–24], all of which associate with Stat3. These docking motifs are generally membrane-proximal presumably because Stat3, upon binding, becomes close to the inner cell membrane, facilitating its activation [25].

## Conclusions

Bladder cancer is the ninth most frequently diagnosed cancer, and thirteenth most common cause of cancer-specific mortality, worldwide [26]. Approximately 75% of patients present with a non-muscle invasive form of the disease at first diagnosis [27], but about 20–25% of these will progress to muscle-invasive disease [28, 29]. As a consequence, one of the main problems today in the clinical management of bladder cancer, is the frequent recurrence and progression of non-invasive lesions. We have previously reported Blcap as a prognostic biomarker for human bladder cancer [1]. In an immunohistochemical study of 120 bladder biopsies, we found a dual behavior of Blcap in bladder tumors. We observed down-regulation of Blcap associated with progression, with up to 51% of invasive tumors displaying loss of Blcap expression, and overexpression of Blcap in up to 20% of all cases, irrespective of tumor stage/grade. The latter presented usually with strong, interspersed nuclear staining and was associated with poorer outcome in patients. To clarify the mechanisms underlying the prognostic effect of nuclear Blcap, and because we had evidence suggesting nuclear localization was associated with a signaling event [1], we took two parallel approaches: on the one hand we profiled the TIF protein content of biopsies for cytokine and growth factor expression (Fig 1), and on the other hand we performed a signal pathway analysis using an immunophenotyping strategy (Fig 4). The expectation was that by combining the results of the two analyses, we would get reciprocal evidence for involvement of a given signaling pathway in nuclear expression of Blcap. We found that expression of three cytokines, MCP-1, IL-8, and IL6, followed nuclear expression of Blcap, and of all the signaling intermediates examined, only p-Stat3 correlated with Blcap. Stat3 is one of a family of transcription-regulating signaling proteins that transduce environmental stimuli, such as cytokines and growth factors, through a signaling pathway, the Janus kinase/signal transducers and activators of transcription (Jak/Stat) pathway [30, 31]. Together, our data indicated that Blcap expression was associated with Jak/Stat signaling. The Jak/Stat pathway regulates key cellular processes such as cell proliferation and survival, and aberrant Jak/Stat signaling has been linked to cancer progression and metastasis [11, 32]. Previous studies have also found that between 19 and 40% of bladder cancers have elevated expression of p-Stat3 [11, 33], a figure comparable to the one we found (20%) for Blcap overexpression in bladder cancer [1]. Given that activated Stat3 drives transcription of a number of genes, the associations we observed could simply be due to the fact that Blcap expression was under control of Stat3. However, this does not appear to be the case. Stimulation of T24 bladder cancer cells with IL-6, leads to Stat3 activation, as determined by phosphorylation of Stat3 (Fig 7, western blot, p-Stat3), but we found no evidence of increased Blcap expression (Fig 7, western blot, Blcap). We also found tissue biopsies with high levels of p-Stat3 expression that were devoid of Blcap (Fig 5). In addition, we found that localization of Blcap was highly correlated to that of p-Stat3 (Figs 2 and 3), suggesting that nuclear presence of Blcap might be the result of a physical interation with Stat3. The presence of a consensus Stat3-docking site, a YXXQ motif, Y^2^CLQ^5^ proximal to one of the transmembrane domains, supports this notion. In fact, we could show, both in tissue samples where we could find evidence of physical proximity (Fig 6), but also in the T24 bladder cancer cell line, where Stat3 activation potentiated interaction of activated Stat3 with Blcap (Fig 7), that Blcap physically interacts with Stat3. This interaction may explain the unexpected finding of nuclear presence of Blcap, as well as the dual behavior we observed for Blcap, with tumors displaying lower levels of expression in of Blcap expression with progression, but patients bearing tumors with strong nuclear expression of Blcap doing worse than those with low to moderate expression [1].

Our observations also raise a number of questions: is there a biological consequence to the interaction? Can binding to Blcap modulate Stat3 activity, and is the interaction necessary for Stat3 function? It would appear that Blcap binding to Stat3 is associated with activation of the latter, and since only the phosphorylated form of Stat3 translocates to the nucleus, it would be tempting to conclude that Blcap binds only to the phosphorylated form of the protein, or alternatively that binding to Blcap stimulates phosphorylation of Stat3. Immunoprecipitation analysis of cellular fractions supports this conclusion, as we could only detect interaction of Blcap with Stat3 in the nuclear fraction.

In conclusion, we have identified Blcap as a new Stat3 interactor in bladder cancer. Several interesting questions now arise and further studies are needed to clarify the role of Blcap in Jak/Stat signaling, and specifically in bladder tumorigenesis. On a final note, there is one additional aspect of Blcap biology that should be looked into in relation to Stat3 signaling, namely that Blcap undergoes multiple A-to-I editing events in bladder cancer [34, 35]. In hepatocellular carcinoma (HCC), Blcap was shown to be a novel editing gene with over-editing expression in approximately 40% HCCs compared to adjacent liver tissues [36]. The RNA-edited form of Blcap was able to promote cell proliferation [36]. As a result it is possible that overediting of Blcap regulates expression of the Stat3 interaction which in turn may play a regulatory role in Stat3-mediated signaling, and ultimately in bladder carcinogenesis.

While we were revising this article, a study was published that not only supports our observations, but also clarifies some of the unusual aspects of the Blcap biology we have reported [37]. In this study, Chen and colleagues showed that Blcap interacted with Stat3 in cervical cancer cell lines, supporting our own observation that Blcap interacts with Stat3 in bladder cancer. In addition, these authors also showed that A-to-I RNA editing of Blcap leads to a loss-of-function of Blcap, affecting its anti-tumorigenic behavior in cervical cancer. Together with our own observations in bladder cancer samples, this data suggests that Blcap interaction with Stat3 may be a general event, and that this interaction has a regulatory role on Stat3 activity and presumably on cancer development. The modulation of Blcap function by A-to-I RNA editing also provides a suggestive mechanism to explain the bimodal effects we observed for Blcap in bladder cancer, and some of the challenging observations we made, concerning loss of expression of Blcap with bladder cancer progression, and the fact that tumors that expressed this protein at very high levels showed a worse prognosis.

## Supporting information

S1 Table List of all antibodies used in this study. (DOC)

S2 Table Clinicopathological characteristics of the samples used in this study. (DOC)
