# Supplementary material for: Identification of BLCAP as a novel STAT3 interaction partner in bladder cancer
Source: PLoS One. 2017 Nov 30;12(11):e0188827. doi: 10.1371/journal.pone.0188827 (PMC5708675; doi:10.1371/journal.pone.0188827)
Supplement: S1 Table — (DOC) [file pone.0188827.s001.doc]

**S1 table.** List of all antibodies used in this study

| *Antigen* | *Dilution* | *Supplier* | *Cat. Nr.* |
| --- | --- | --- | --- |
| Blcap | 1:500 | Eurogentec | - |
| Lamin A | 1:1000 | Santa Cruz Biotechnology | sc-71481 |
| GAPDH | 1:5000 | Cell Signaling Technologies | #5174 |
| p38 MAPK (pThr180,Tyr182) | 1:150 | Cell Signaling Technologies | 4631 |
| p44/42 MAPK (pThr202/185,Tyr204/187) | 1:150 | Cell Signaling Technologies | 9101 |
| Sapk/Jnk (pThr183/Tyr185) | 1:150 | Cell Signaling Technologies | 4668 |
| Akt (pSer473) | 1:100 | Invitrogen (Biosource) | 44-621G |
| GSK-3-β (pSer9) | 1:250 | Cell Signaling Technologies | 9322 |
| Stat3 (pTyr705) | 1:150 | Cell Signaling Technologies | 9131 |
| Stat1 (pTyr701) | 1:150 | Cell Signaling Technologies | 9171 |
| p53 (pSer15) | 1:250 | Cell Signaling Technologies | 9284 |
| p53 (pSer392) | 1:250 | Cell Signaling Technologies | 9281 |
| Ki67 | 1:1000 | Becton Dickinson | 610968 |
| mTor (pSer2448) | 1:100 | Cell Signaling Technologies | 2971 |
| Atm (pSer1981) | 1:400 | Rockland Immunochemicals | 200-301-400 |
| Chk2 (pThr68) | 1:250 | Cell Signaling Technologies | 2197 |
| Rb (pSer807/811) | 1:200 | Cell Signaling Technologies | 8516 |
| Cleaved Caspase-3 (Asp175) | 1:80 | Cell Signaling Technologies | 9664 |
| Stat6 (Tyr641) | 1:150 | Cell Signaling Technologies | 9364 |
| HER2/ErbB2 (Tyr1221/1222) | 1:150 | Cell Signaling Technologies | 2243 |
| EGF Receptor (Tyr1068) | 1:150 | Cell Signaling Technologies | 2234 |
| EGF Receptor (Tyr992) | 1:150 | Cell Signaling Technologies | 2235 |
| EGF Receptor (Tyr845) | 1:150 | Cell Signaling Technologies | 2231 |
| EGF Receptor (Tyr1045) | 1:150 | Cell Signaling Technologies | 2237 |
