# Supplementary material for: Identification of BLCAP as a novel STAT3 interaction partner in bladder cancer
Source: PLoS One. 2017 Nov 30;12(11):e0188827. doi: 10.1371/journal.pone.0188827 (PMC5708675; doi:10.1371/journal.pone.0188827)
Supplement: S2 Table — (DOC) [file pone.0188827.s002.doc]

**S2 table.** Clinicopathological characteristics of the samples used in this study

| *Sample* | *Grade* | *Stage* |
| --- | --- | --- |
| T#1 | G2 | Ta |
| T#2 | G3 | T1 |
| T#3 | G3 | T2-4 |
| T#4 | G3 | T2-4 |
| T#5 | G3 | T1 |
| T#6 | G3 | T1 |
| T#7 | G3 | T1 |
| T#8 | G3 | T2-4 |
| T#9 | G2 | Ta |
| T#10 | G3 | T2-4 |
